# Integration of imaging and clinical biomarkers for cerebral infarction diagnosis via NeuroFusionNet

**DOI:** 10.3389/fnins.2026.1759771

**Published:** 2026-07-22

**Authors:** Jun Zhao, Yongkun Gui, Lingling Zhang, Feixiang Li, Dewei Zhu, Haoliang Wang, Yuhua He, Ping Zhang

**Affiliations:** 1 Department of Neurology, The First Affiliated Hospital of Henan Medical University, Xinxiang, Henan, China; 2 Henan Medical Key Laboratory of Neurology, The First Affiliated Hospital of Henan Medical University, Xinxiang, Henan, China; 3 Henan Joint International Laboratory of Neurorestoratology for Senile Dementia, The First Affiliated Hospital of Henan Medical University, Xinxiang, Henan, China; 4 Henan Key Laboratory of Neurorestoratology and Protein Modification, The First Affiliated Hospital of Henan Medical University, Xinxiang, Henan, China; 5 Department of Rehabilitation, The First Affiliated Hospital of Henan Medical University, Xinxiang, Henan, China; 6 Department of Radiology, The First Affiliated Hospital of Henan Medical University, Xinxiang, Henan, China

**Keywords:** cerebral infarction, clinical biomarkers, medical image analysis, multi–modal deep learning, neural network fusion

## Abstract

**Background:**

Cerebral infarction remains a leading cause of mortality and long-term disability worldwide, demanding rapid and accurate diagnostic strategies. However, current assessments primarily rely on imaging interpretation, often neglecting valuable clinical and laboratory information that could enhance diagnostic precision.

**Methods:**

We developed NeuroFusionNet, a multi-modal deep learning framework that integrates imaging features with clinical biomarkers for binary classification of cerebral infarction and healthy controls. The model combines a ResNet-based visual encoder with a multilayer perceptron branch for clinical indicators, achieving end-to-end feature fusion and joint optimization.

**Results:**

NeuroFusionNet achieved superior diagnostic performance with an accuracy of 0.9655, precision of 0.9584, recall of 0.9584, and F1-score of 0.9584, significantly outperforming baseline models including ResNet, MobileNet, and GhostNet. The integration of imaging and clinical biomarkers effectively enhanced model sensitivity and robustness, demonstrating strong potential for real-world clinical application.

**Conclusion:**

Our findings highlight the clinical value of integrating imaging and laboratory data for precision diagnosis of cerebral infarction. NeuroFusionNet provides a scalable and interpretable framework that may support early detection and personalized management of cerebrovascular diseases in routine clinical practice.

## 1 Introduction

**Figure 1:**
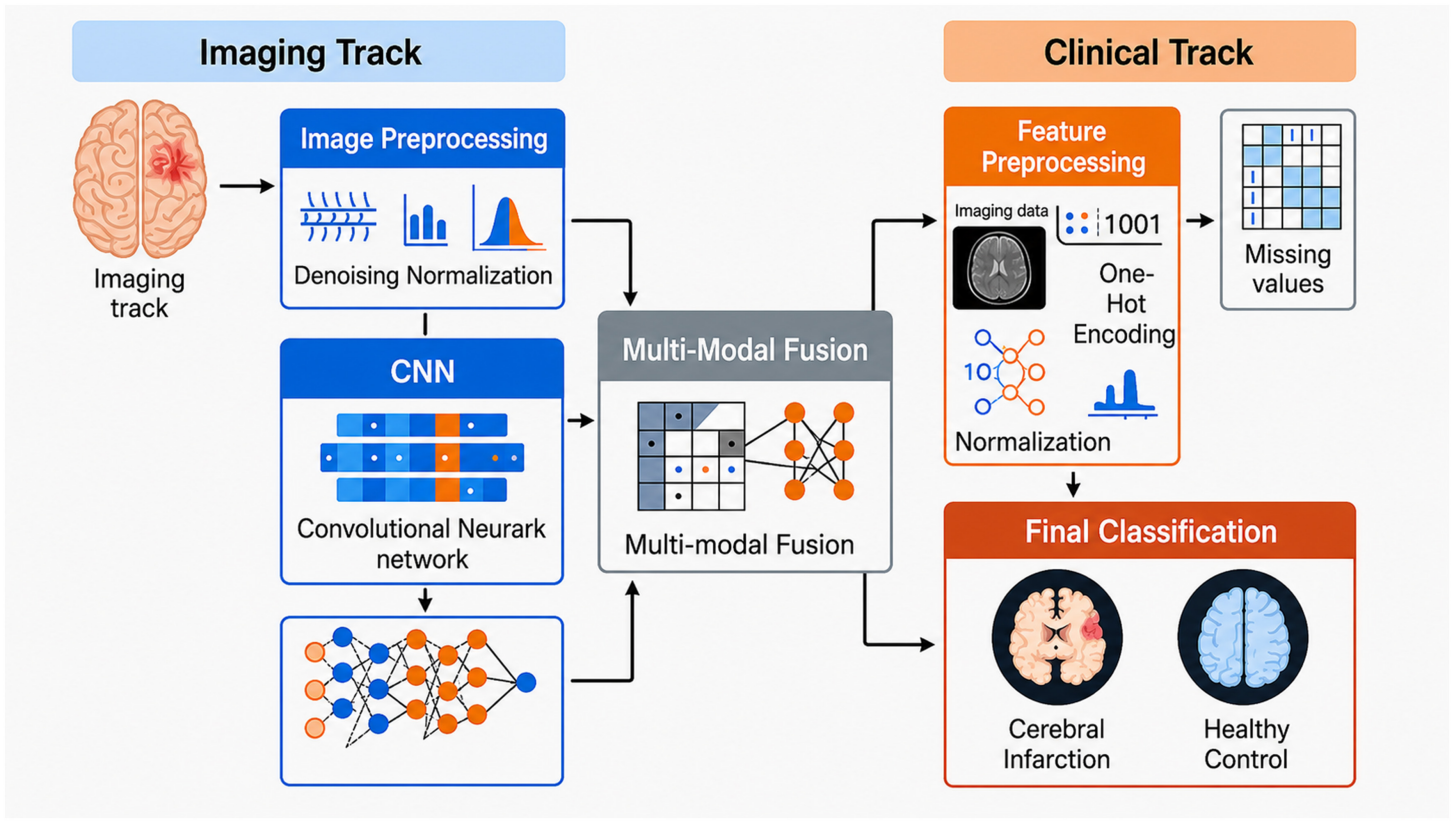
Overview of the NeuroFusionNet multimodal diagnostic workflow. The pipeline consists of two coordinated branches: an imaging track, where brain scans undergo denoising, normalization, and convolutional feature extraction through a ResNet-based encoder, and a clinical track, where laboratory indicators are processed through imputation, one-hot encoding, and normalization before being embedded via a multilayer perceptron. The resulting high-level representations are fused within a dedicated multimodal integration module, enabling joint learning across heterogeneous data sources. The fused features are subsequently fed into a final classification head to output the probability of cerebral infarction vs. healthy control.

Ischemic stroke (cerebral infarction) is a leading cause of mortality and long-term neurological disability worldwide, accounting for more than two-thirds of all stroke events and contributing substantially to the global burden of cerebrovascular disease (Li et al., 2024; Feigin et al., 2025; Liu S. et al., 2025). Despite progress in preventive strategies and acute interventions, the absolute incidence of ischemic stroke continues to rise, driven by population aging, the growing prevalence of modifiable vascular risk factors, and rapid demographic transitions (Fan et al., 2023; Cheng et al., 2024). Survivors frequently experience persistent motor, cognitive, and functional impairments, and the risk of recurrent ischemic events remains considerable, with 5-year recurrence rates exceeding 10% across multiple contemporary cohorts (Verburgt et al., 2024; Boulanger et al., 2018). In parallel, the economic impact of ischemic

stroke remains profound, encompassing escalating healthcare expenditures, prolonged rehabilitation requirements, and substantial long-term care burdens across diverse health systems (Imoisili, 2024; He et al., 2024; Quinn et al., 2025). Despite the clear clinical urgency, diagnosing cerebral infarction remains challenging in practice, largely due to the high demand on physician expertise, variations in interpretation, and workflow inefficiencies. Radiologists and neurologists must integrate complex imaging patterns with subtle clinical and laboratory signals under tight time constraints, which increases the susceptibility to human error and inter-observer variability (Bojsen et al., 2024; Fernandes et al., 2024). Moreover, training of specialized stroke neuroimagers is resource-intensive, the manual review of large volumes of scans is time-consuming, and the turnaround times often hinder early intervention (Adablanu et al., 2025; Al-Janabi et al., 2024). Lesion boundaries may be subtle, anatomical variants misleading, and small vessel occlusions frequently overlooked—all of which contribute to diagnostic delays and misclassification (Liu et al., 2024). Given these limitations, there is an urgent need for fully automated, high-throughput, assistive tools that reliably triage and detect infarctions, thereby reducing both workload and diagnostic latency. In this context, our proposed multi-modal framework, NeuroFusionNet, aims to bridge the gap by fusing imaging features with clinical biomarkers to deliver rapid, accurate, and scalable cerebral infarction detection, addressing the current bottlenecks in clinical workflow and decision-support systems (Zhao et al., 2024; Baaklini and Valdés Hernández, 2025; Kousar et al., 2025). With the rapid evolution of artificial intelligence, deep learning–based methods have increasingly been applied to the detection and classification of cerebral infarction. One study trained a convolutional neural network on diffusion-weighted MRI to classify ischemic stroke territory at the patient level, demonstrating promising discrimination performance (Ryu et al., 2024). Another investigation developed a deep learning algorithm for ischemic stroke subtype classification using DWI and atrial fibrillation data across multiple centers (Jung et al., 2024). An advanced model achieved 84% accuracy in differentiating ischemic stroke using MRI scans and pretrained ConvNeXt architecture (Jiang et al., 2025). A further report introduced a multimodal approach combining MRI, CT and structured clinical data to predict stroke recurrence with improved accuracy over imaging only models (Fan et al., 2024). Nevertheless, substantial challenges remain: most existing classifiers rely exclusively on single-modality input (Oliveira et al., 2023); Ou et al., 2025). In addition, many studies focus on segmentation or lesion delineation rather than direct patient-level binary classification of infarct vs. healthy controls, which restricts translational potential in routine clinical workflows (Heo, 2025). These gaps underscore the imperative for a fully integrated multi-modal classification framework that unifies imaging and biomarker data in an end-to-end deep learning model. Accordingly, we present NeuroFusionNet, designed to fuse imaging features with laboratory-clinical indicators and thereby overcome the limitations of current models in cerebral infarction detection. In this study, we introduce NeuroFusionNet, a multi-modal deep learning framework designed to enhance the detection of cerebral infarction by integrating imaging features with laboratory and clinical biomarkers in an end-to-end architecture. The model combines a ResNet-based visual encoder with a multilayer perceptron branch for structured clinical indicators, enabling joint feature learning and cross-modal representation fusion at the patient level. This unified approach is specifically tailored for binary classification of infarction vs. healthy controls, avoiding the need for lesion annotations and thus improving clinical scalability. The key innovations of this work include: (i) a fully automated multi-modal fusion strategy that leverages heterogeneous sources of patient data; (ii) an end-to-end optimization pipeline that enables synergistic learning between imaging and biomarker domains; (iii) a lightweight, high-efficiency architecture capable of delivering rapid inference suitable for real-time clinical workflows; and (iv) comprehensive evaluation across multiple baseline models to demonstrate superior robustness and diagnostic performance. Collectively, NeuroFusionNet seeks to address long-standing limitations in single-modality systems and provide a more accurate, interpretable, and clinically actionable tool for cerebral infarction detection. Figure 1 provides a schematic workflow of the proposed NeuroFusionNet framework.

## 2 Method

### 2.1 Data acquisition

**Figure 2:**
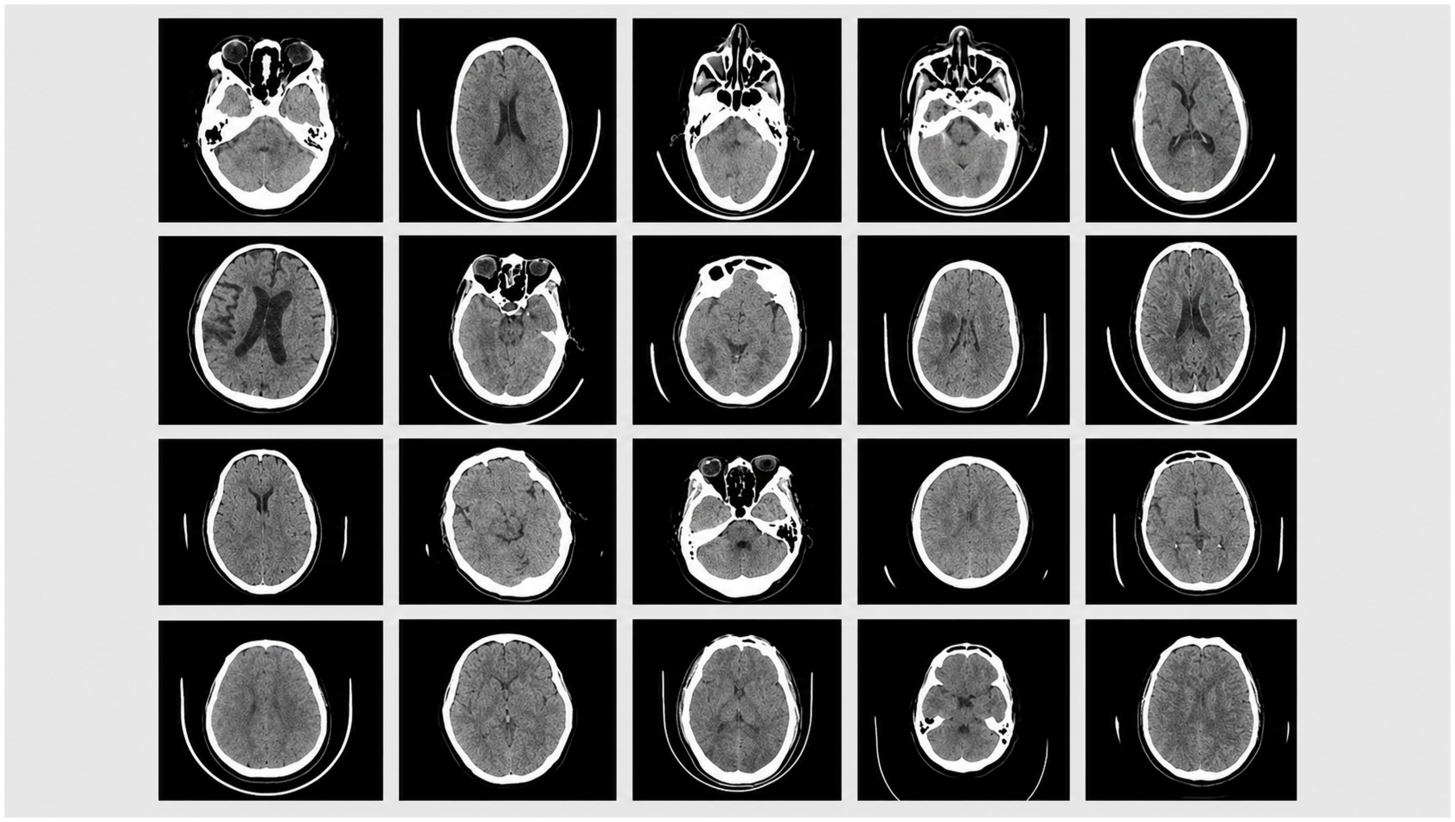
Representative non-contrast head CT slices from the study cohort. Examples are shown from both cerebral infarction cases and healthy controls, illustrating typical appearance variability across patients and slice levels.

This study was reviewed and approved by the Ethics Committee of The First Affiliated Hospital of Xinxiang Medical University (Approval No. EC-2025-704). All data used in this study were retrospectively collected from the First Affiliated Hospital of Xinxiang Medical University between July 1, 2023, and January 31, 2025. A total of 248 subjects were enrolled following strict inclusion and exclusion criteria (Table 1), consisting of 191 patients with acute ischemic cerebral infarction confirmed by MR-DWI and 57 healthy controls without neurological abnormalities. All participants underwent non-contrast head CT and/or MR-DWI within 48 h of presentation, and infarction cases satisfied the clinical requirement of symptom onset to imaging less than 24 h, with a CT–MR interval not exceeding 6 h. Eligible individuals were required to be over 18 years old and capable of completing at least one qualifying imaging modality. Patients were excluded if they were pregnant, had recently participated in high-risk interventional trials, had severe uncontrolled systemic disease, or exhibited imaging sequences that could not be reliably evaluated. In addition to imaging, every enrolled subject provided a complete set of laboratory biomarkers at admission, including hematological, biochemical, endocrine, and coagulation indicators. These structured clinical measurements formed a parallel modality that enabled construction of a fully integrated multimodal dataset, supporting joint imaging–biomarker feature learning in the proposed NeuroFusionNet framework. Meanwhile, Figure 2 presents some of our image dataset.

**Table 1:** Inclusion and exclusion criteria used to define the study cohort.

Category	Criteria
Inclusion criteria	1. Age ≥ 18 years.
	2. Clinically suspected or confirmed acute ischemic stroke (first-ever or recurrent).
	3. Time from symptom onset to imaging examination (CT or MR-DWI) ≤ 24 h.
	4. Completion of at least one imaging modality (non-contrast CT or MR-DWI); CT–MR interval allowed ≤ 6 h.
	5. Availability of complete laboratory biomarker panel at admission.
Exclusion criteria	1. Pregnancy.
	2. Participation in high-risk interventional clinical trials within the past 3 months.
	3. Severe uncontrolled systemic disease (e.g., end-stage cardiac, hepatic, or renal failure; active malignancy).
	4. Non-diagnostic imaging due to severe artifacts or missing essential sequences.

### 2.2 Data preprocessing

All imaging and clinical data were processed through a unified preprocessing workflow to standardize input quality and enhance model stability. For imaging data, raw scans were first screened to remove corrupted files, incomplete slices, or acquisition artifacts. A hybrid Gaussian–median filtering strategy was then applied to suppress high-frequency noise while preserving lesion-related structural boundaries. Intensity normalization and contrast-limited adaptive histogram equalization (CLAHE) were used to correct illumination variability and improve contrast in infarct-relevant regions. To reduce scanner-dependent heterogeneity, all images were resampled to a fixed spatial resolution and center-cropped to retain diagnostically relevant brain regions. During training, extensive data augmentation—including random rotations (±15°), flips, affine transformations, elastic deformations, random cropping, and contrast jittering—was applied to simulate realistic anatomical and acquisition variability. Minority-class oversampling combined with augmentation-based synthetic

expansion was further used to balance infarction and healthy-control samples, resulting in a more diverse and representative training set. All subjects underwent non-contrast head CT scanning as part of routine clinical care within the acute time window after admission. CT images were converted into axial 2D slices for model training. For each patient, we selected 20–30 representative axial slices covering the brain parenchyma (excluding slices dominated by non-brain background) to ensure consistent patient-level representation. Each slice was resized to 224 × 224 pixels. CT intensities were processed using a fixed brain-window setting (e.g., WL/WW = 40/80) and then normalized to [0,1] before being fed into the network. Data augmentation was applied only on the training split/folds, including random horizontal flip (*p* = 0.5), random rotation (±10°), and mild affine transformations (translation up to 5%, scale 0.95–1.05). To address class imbalance (191 infarction vs. 57 controls), imbalance handling was performed only within the training split/folds, using class-weighted cross-entropy loss together with minority-class oversampling and on-the-fly augmentation, to reduce bias toward the majority class.

We employed a 2D ResNet-50 backbone to encode each CT slice and output a slice-level probability. Patient-level predictions were obtained by aggregating slice-level outputs using mean pooling across the selected slices:


$$\begin{array}{l} P=\frac{1}{N}\overset{N}{\underset{i=1}{\sum }}p_{i}, \end{array}$$ (1)

where *p*_*i*_ denotes the predicted probability for the *i*-th slice and *N* is the number of selected slices per patient (20–30 in this study). Since CT is single-channel, each slice was replicated to three channels to match the standard ResNet-50 input interface. The network was initialized with ImageNet-pretrained weights and fine-tuned end-to-end on our dataset.

Structured clinical and laboratory indicators underwent a dedicated preprocessing pipeline to ensure numerical consistency and facilitate fusion with imaging features. Each variable was evaluated for completeness: features with < 10% missing values were imputed using median statistics, while those exceeding this threshold were imputed using a k-nearest-neighbor (k-NN) approach to preserve nonlinear inter-variable dependencies. Continuous variables were normalized using z-score standardization to unify the scale across heterogeneous biomarkers such as inflammatory markers, metabolic indices, and serum chemistry results. Categorical variables were transformed using one-hot encoding. Outlier detection based on the interquartile range (IQR) method was applied, with values beyond 3 × IQR clipped or winsorized to mitigate measurement errors and extreme anomalies. Missing-value handling and feature transformations were implemented in a strictly leakage-free manner. Specifically, for each training fold, imputation parameters were learned using only the training portion and then applied to the corresponding validation/test portion. Variables with < 10% missingness were imputed with the training-fold median, whereas variables with higher missingness were imputed using k-nearest-neighbor imputation (*k* = 5, Euclidean distance). Continuous variables were standardized using z-score normalization with mean and standard deviation computed from the training fold only. Categorical variables (e.g., urinalysis semi-quantitative items) were encoded via one-hot representation based on categories observed in the training fold. All structured variables listed in Table 2 were used as inputs to the clinical branch.

**Table 2:** Structured laboratory and clinical variables used as model inputs.

Variable	Type	Unit	Summary	Missing (%)
P-LCR	Continuous	%	27.15 ± 6.86 (range 11.90–47.60)	8.1
TSH	Continuous	mIU/L	2.24 ± 1.57 (range 0.04–8.19)	11.1
HCY	Continuous	μmol/L	18.35 ± 11.66 (range 6.20–57.95)	54.0
ZWXB	Categorical	a.u.	0 (97.4%)	41.4
GLU	Categorical	a.u.	Negative (53.3%)	1.5
TS	Categorical	a.u.	None (100.0%)	28.8
YS	Categorical	a.u.	Yellow-brown (100.0%)	28.8
TP	Continuous	g/L	64.33 ± 7.52 (range 47.30–91.90)	6.1
ZT	Categorical	a.u.	Soft (100.0%)	28.8
MCH	Continuous	pg	30.96 ± 2.12 (range 23.00–38.93)	8.1
JSYC	Categorical	a.u.	0 (97.7%)	33.8
OB	Categorical	a.u.	Negative (85.7%)	28.8
SG	Categorical	–	1.02 (31.1%)	10.6
JDZZ	Categorical	a.u.	0 (97.7%)	33.8
NXB	Categorical	a.u.	0 (92.9%)	28.8
PDW	Continuous	fL	11.78 ± 1.88 (range 8.10–17.30)	9.1
MJ	Categorical	a.u.	0 (97.7%)	33.8
XKLDJJ	Categorical	a.u.	0 (97.7%)	33.8
JBYJ	Categorical	a.u.	0 (97.7%)	33.8
MALB	Categorical	a.u.	Negative (71.4%)	10.6

Continuous variables are summarized as mean ± SD with ranges after applying the same preprocessing used for modeling (including outlier clipping). Categorical variables are summarized by the most frequent category (percentage among non-missing). Missingness is reported as the proportion of subjects without an available value.

Following preprocessing, all clinical features were concatenated into a unified structured vector and paired with the processed imaging tensors, providing NeuroFusionNet with two complementary and consistently formatted data streams. This integrated preparation ensures that the model receives noise-reduced, contrast-enhanced imaging inputs alongside statistically coherent clinical biomarkers, enabling stable optimization and effective cross-modal feature fusion in the subsequent learning pipeline. Importantly, imputation statistics (median/k-NN), feature scaling parameters (mean/standard deviation), and any class-balancing strategy were computed using training data only within each cross-validation fold, and then applied to the corresponding validation fold and the independent test set. Data augmentation was performed only for training samples and was never applied to validation or test data.

### 2.3 Construction of model

#### 2.3.1 Proposed deep learning model

In recent years, deep convolutional neural networks and derived architectures have been increasingly applied to ischemic stroke detection and classification. For example, a comprehensive survey by Cui et al. (2022) reviewed major applications of deep learning in acute ischemic stroke imaging, including segmentation and classification tasks across MRI modalities. Zhou et al. developed a machine learning-based scoring model for predicting mortality in ICU-admitted ischemic stroke patients with moderate to severe consciousness disorders (Zhou et al., 2025). Aksoy et al.(2024) employed a ConvNeXt-based network to classify ischemic strokes on MRI, achieving an overall accuracy of around 84% in a test set, illustrating the potential of modern architectures for stroke prediction. Koska et al. (2025) trained pretrained MobileNetV2 and EfficientNet B0 models on DWI images for patient-level classification of ischemic stroke territories, achieving 0.95 accuracy in slice-wise classification but noted accuracy dropped to 0.88 on external testing. Kulathilake et al. (2025) reported a multi-class deep learning classification framework on CT images of brain stroke patients, demonstrating high precision and F1-scores in retrospective cohorts. Tsai et al. proposed a fusion model combining DWI -ADC images with structured health-profile data using contrastive learning and achieved an AUC of 0.87 for functional-outcome prediction, demonstrating the value of multimodal inputs while remaining focused on prognosis rather than binary classification of cerebral infarction (Tsai et al., 2024). Additional works show similar patterns: despite strong performance in experimental settings, many algorithms remain limited to segmentation tasks or single-modality inputs, and external generalization remains weak (Bayram et al., 2025). These limitations—including dependence on lesion delineation, single-modality training, decreasing accuracy in cross-center validation, and limited focus on binary classification of infarct vs. healthy control—motivate the development of a new multi-modal classification architecture. Against this backdrop, we introduce NeuroFusionNet, specifically designed to fuse imaging and clinical biomarker data for robust patient-level binary classification of cerebral infarction.

Building upon these observations, NeuroFusionNet was designed as a unified multi-modal architecture that integrates imaging representations with structured clinical biomarkers in a single end-to-end framework. The imaging branch employs a ResNet-50 backbone pretrained on ImageNet, selected for its strong balance between representational capacity and computational efficiency. After the standard convolutional and residual blocks, the final feature tensor is processed through a squeeze-and-excitation (SE) channel recalibration module to enhance discriminative responses in channels most relevant to infarction patterns. The encoder output is globally averaged pooled and flattened to form a 2048-dimensional imaging feature vector, capturing both low-level structural cues (e.g., intensity transitions, tissue boundaries) and high-order semantic patterns characteristic of infarction.

Parallel to this, structured clinical indicators—including laboratory biomarkers and physiological measurements—are processed through a multilayer perceptron (MLP) branch. This branch consists of two fully connected layers comprising 256 and 128 neurons, respectively, each followed by batch normalization and ReLU activation to stabilize training across heterogeneous clinical variables. A dropout layer (rate = 0.3) is incorporated to reduce overfitting, particularly given the varied distribution of laboratory indices. The final clinical embedding is projected into a 128-dimensional latent vector, ensuring compatibility with the imaging pathway.

To achieve effective cross-modal integration, NeuroFusionNet concatenates the 2,048-dimensional imaging vector with the 128-dimensional clinical embedding, yielding a fused feature vector that is passed through a joint fusion module. The 128-dimensional clinical embedding was produced from the full structured input vector (Table 2) after fold-wise imputation and standardization, ensuring that the clinical modality is on a comparable numerical scale before fusion. This module comprises a 512-unit fully connected layer with ReLU activation, followed by 50% dropout and a final sigmoid classification neuron for binary prediction. The full model contains approximately 27.4 million trainable parameters, with 23 million derived from the ResNet backbone and the remainder from the clinical and fusion pathways. Training is conducted using the AdamW optimizer (learning rate = 1 × 10^−4^, weight decay = 1 × 10^−5^) with a mini-batch size of 32, cosine-annealing learning rate scheduling, warm-up initialization, and early stopping (patience = 15). This design enables NeuroFusionNet to leverage complementary information from both modalities, improving robustness, generalization, and diagnostic fidelity in comparison to single-modality baselines.

**Figure 3:**
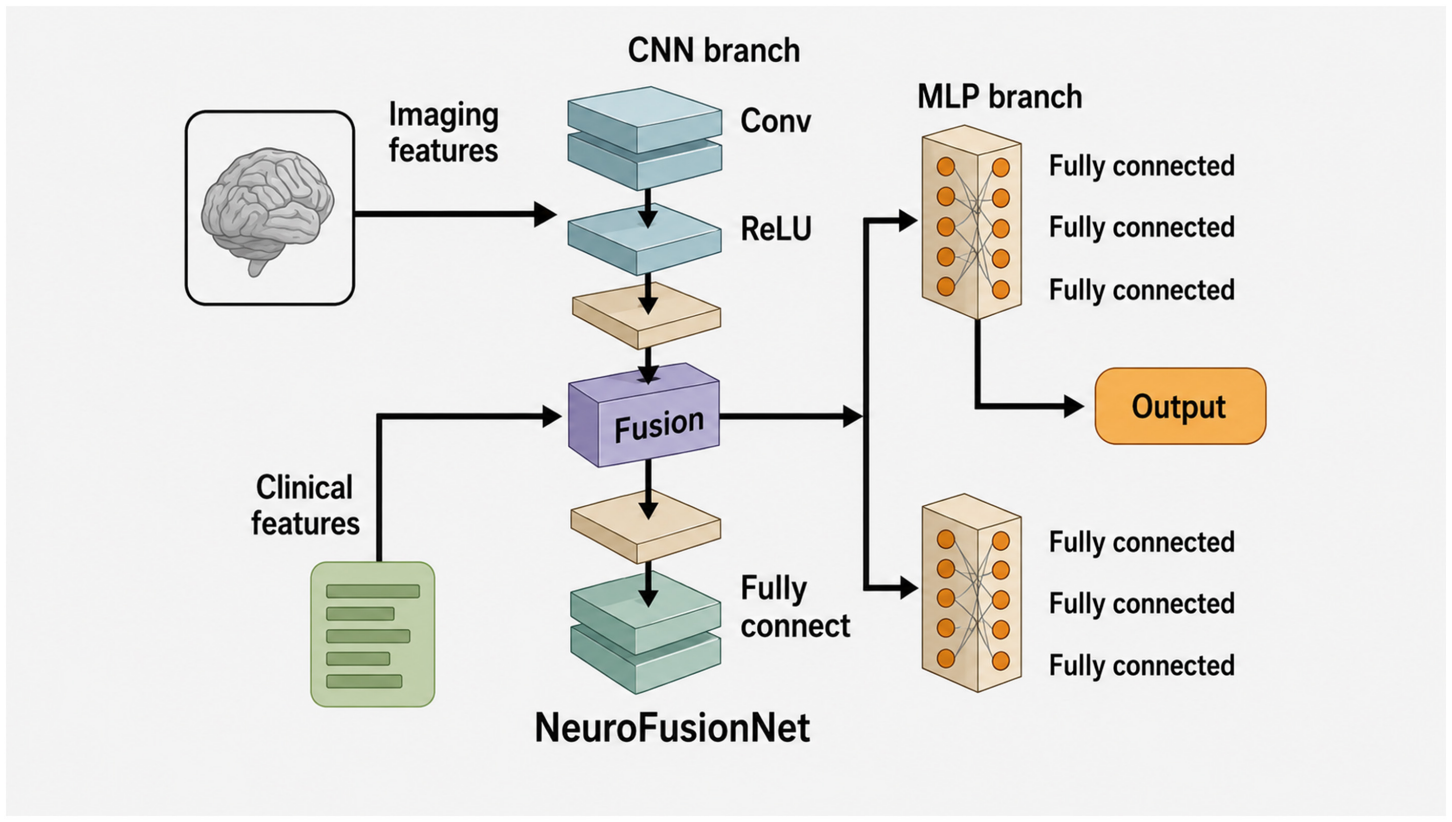
Architectural overview of the proposed NeuroFusionNet model.The framework consists of two parallel branches designed to extract complementary representations from heterogeneous data sources. The imaging branch employs a convolutional neural network (CNN) to hierarchically encode spatial features from brain scans through stacked convolution, activation, and fully connected layers. In parallel, the clinical branch processes structured laboratory and physiological indicators using a multilayer perceptron (MLP), enabling the extraction of high-level semantic embeddings from non-imaging variables. A dedicated fusion module integrates the latent representations from both streams, enabling cross-modal interaction and enhancing the model's ability to capture pathophysiological signatures associated with cerebral infarction. The fused feature vector is subsequently passed to a fully connected classification head that outputs the probability of infarction vs. healthy control.

**Figure 4:**
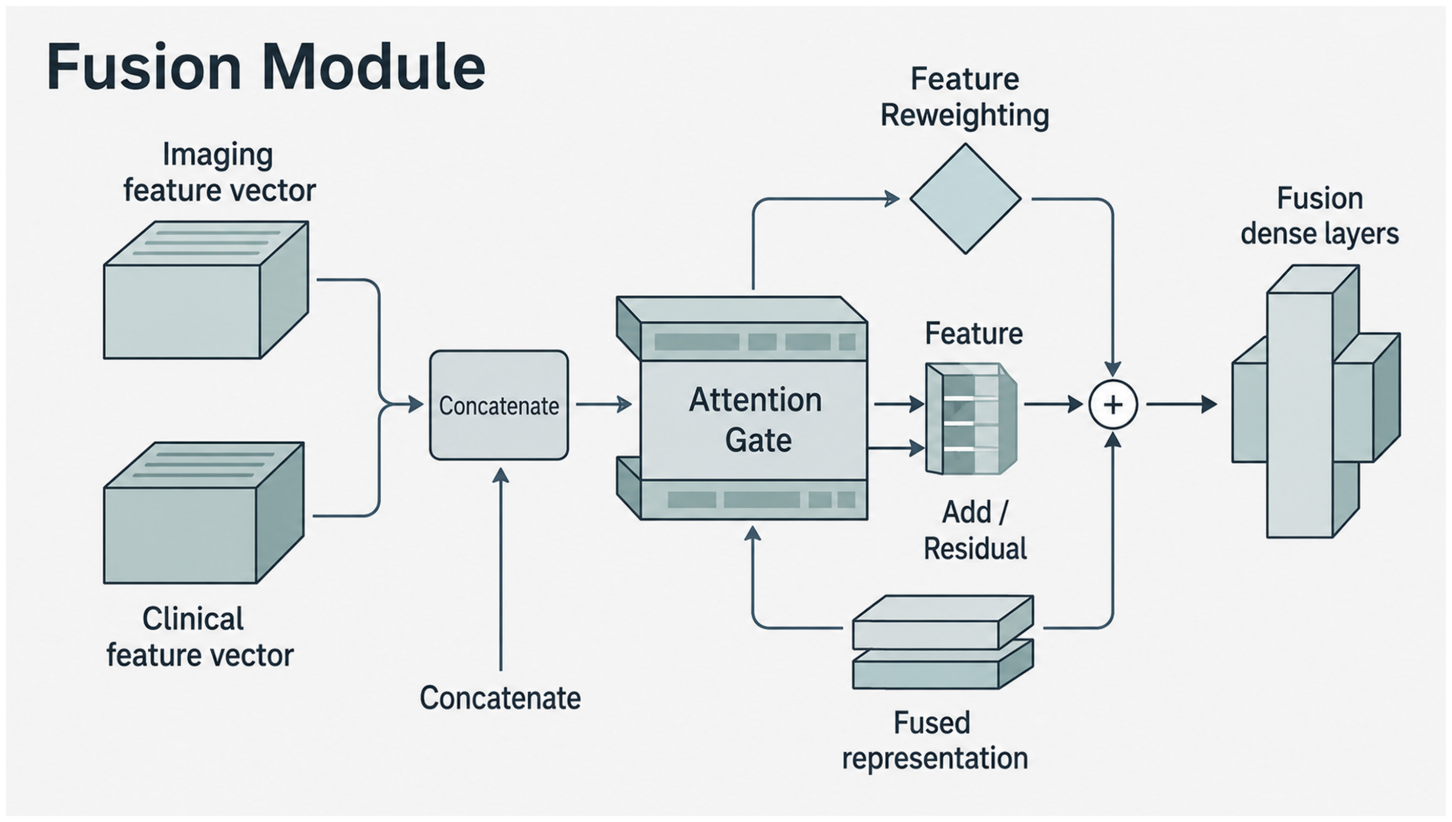
Fusion module architecture of NeuroFusionNet. The imaging and clinical feature vectors are concatenated and passed through an attention-based gating mechanism for adaptive feature reweighting. A residual enhancement block refines the joint representation before projection into fusion-dense layers, generating the integrated feature space used for final classification.

Building on this architecture, NeuroFusionNet offers several features that directly address the challenges inherent to cerebral infarction detection. By combining anatomically rich imaging representations with physiologically informed clinical biomarkers within a single fused representation, the model is able to detect subtle ischemic changes that may be visually inconspicuous or confounded by normal anatomic variability. The integrated modality design allows clinical indicators—such as inflammatory, metabolic, or coagulation-related biomarkers—to reinforce or disambiguate imaging features, thereby improving discrimination between true infarction and benign mimics. In parallel, the patient-level, annotation-free formulation eliminates the dependence on manual lesion delineation, which is particularly advantageous in stroke care where precise contours are rarely available in routine practice. The lightweight fusion head and optimized training strategy further support rapid inference and stable generalization across heterogeneous scanners and patient populations. The detailed architectural design of NeuroFusionNet is illustrated in Figures 3, 4.

#### 2.3.2 Comparative models

In our benchmarking study, we selected five representative architectures as comparative baselines to contextualize the performance of our multi-modal framework. ResNet is a deep residual network that introduced skip-connections to ease degradation and enable very deep models; Tsai et al., 2024). GhostNet is an efficient architecture that generates “ghost” Xue et al., 2025). MobileNet, with its depthwise separable convolutions tailored for mobile and edge deployment, has likewise been used in brain MRI/CT classification workflows where real-time inference is prioritized (Elsayed et al., 2025). RegNet, a family of networks derived from design-space rules that trade-off scalability and efficiency, has been applied in medical image classification though not yet widely in cerebral infarction detection (Ekingen et al., 2025). Finally, ShuffleNet, engineered for ultra-low compute environments via channel-shuffle and group convolution, offers extreme efficiency but tends to sacrifice sensitivity—an effect evident in our experiments when applied to infarction vs. control classification. Taken together, while each of these models brings distinct architectural strengths, our results suggest that purely imaging-based, single-modality pipelines still struggle to capture the full complexity of cerebral infarction detection—hence the impetus for our multi-modal solution.

### 2.4 Experimental setup

All experiments were conducted under a standardized and reproducible training protocol. All data splitting was performed at the patient level to prevent subject-wise leakage. We first created an independent hold-out test set (20%) using stratified sampling to preserve the infarction/control ratio; the test set was kept untouched until the final evaluation. The remaining 80% of patients constituted the development set, on which we conducted stratified five-fold cross-validation for model selection (hyperparameter tuning and early stopping). For each fold, preprocessing steps that require parameter estimation were fitted using the training fold only and then applied to the corresponding validation fold. After selecting the final configuration, the model was retrained on the full 80% development set and evaluated once on the independent test set. We fixed the random seed (seed = 2025) for splitting and model initialization to ensure reproducibility, and we report the number of folds (*k* = 5) and the hold-out ratio explicitly. Data augmentation was applied only to the training data during model fitting, and no slice/patch-level mixing across patients was allowed in any split. This unified experimental environment ensures that performance differences among models arise from architectural characteristics rather than confounding factors related to hardware, software, or hyperparameter choices.

### 2.5 Model evaluation

To assess model performance in cerebral infarction detection, we adopted four widely used evaluation metrics: accuracy, precision, recall, and F1-score. Accuracy quantifies the overall proportion of correctly classified subjects, while precision reflects the reliability of positive predictions by measuring how many identified infarction cases are true infarctions. Recall serves as an indicator of the model's sensitivity in detecting infarction, capturing its ability to identify true positive cases even when lesion presentation is subtle. The F1-score, defined as the harmonic mean of precision and recall, provides a balanced measure that accounts for both false positives and false negatives. In addition to accuracy and ROC-AUC, we reported patient-level sensitivity, specificity, PPV, and NPV derived from the confusion matrix. We also performed precision–recall (PR) analysis and reported AUPRC to better characterize performance under class imbalance. Probability calibration was assessed using the Brier score and reliability analysis, and we additionally report ECE as a summary calibration error metric. Together, these complementary metrics offer a comprehensive assessment of diagnostic performance and allow robust comparison between single-modality baselines and our multi-modal NeuroFusionNet. For uncertainty quantification, we estimated 95% confidence intervals (CIs) for AUC and accuracy on the independent test set using bootstrap resampling (1,000 iterations). For AUC comparisons between NeuroFusionNet and each image-only baseline, we used the DeLong test and report two-sided *p*-values. All statistical tests were conducted at the patient level. The corresponding formulas are:


$$\begin{array}{l} \text{Precision}=\frac{\text{TP}}{\text{TP}+\text{FP}} \end{array}$$ (2)

$$\begin{array}{l} \text{Accuracy}=\frac{\text{TP}+\text{TN}}{\text{TP}+\text{TN}+\text{FP}+\text{FN}} \end{array}$$ (3)

$$\begin{array}{l} \text{Recall}=\frac{\text{TP}}{\text{TP}+\text{FN}} \end{array}$$ (4)

$$\begin{array}{l} F1=2\times \frac{\text{Precision}\times \text{Recall}}{\text{Precision}+\text{Recall}} \end{array}$$ (5)

## 3 Results

Across the full set of experiments, NeuroFusionNet demonstrated a consistently superior performance compared with all image-only baseline models, confirming the substantial value of integrating laboratory biomarkers with imaging features. NeuroFusionNet demonstrated consistently superior performance on the independent test set. We further report 95% confidence intervals (bootstrap resampling) and DeLong tests for AUC comparisons to support the reliability of the observed performance gaps. As summarized in Table 3, NeuroFusionNet achieved the highest accuracy (0.9655), recall (0.9584), precision (0.9584), and F1-score (0.9584), outperformed conventional convolutional architectures such as ResNet, MobileNet, GhostNet, RegNet, and ShuffleNet. Even the strongest single-modality baseline (MobileNet) reached only moderate performance (accuracy 0.7241; F1-score 0.6234), while lightweight models exhibited pronounced sensitivity loss (recall = 0.50) and poor F1-scores (0.4141), highlighting the inherent diagnostic limitations of using imaging alone. The ablation analysis of modality and fusion configurations is summarized in Table 4.

**Table 3:** Performance comparison of image-only baselines and NeuroFusionNet on the independent test cohort.

Model	Acc	Recall	F1-score	Precision	AUC	*p*-value
ResNet	0.7586 (0.702–0.810)	0.6055 (0.535–0.675)	0.6074 (0.540–0.675)	0.7774 (0.715–0.830)	0.7446 (0.680–0.805)	1.1 × 10^−6^
GhostNet	0.7069 (0.647–0.767)	0.5000 (0.430–0.570)	0.4141 (0.350–0.490)	0.3534 (0.280–0.430)	0.6485 (0.575–0.720)	4.6 × 10^−8^
MobileNet	0.7241 (0.665–0.782)	0.6155 (0.545–0.685)	0.6234 (0.560–0.690)	0.6557 (0.590–0.720)	0.7475 (0.685–0.810)	3.2 × 10^−5^
RegNet	0.7069 (0.635–0.755)	0.5000 (0.430–0.570)	0.4141 (0.340–0.480)	0.3534 (0.290–0.440)	0.3816 (0.300–0.465)	2.0 × 10^−12^
ShuffleNet	0.7069 (0.645–0.765)	0.5000 (0.430–0.570)	0.4141 (0.340–0.480)	0.3534 (0.270–0.420)	0.3917 (0.310–0.475)	1.1 × 10^−11^
**NeuroFusionNet**	**0.9655 (0.940–0.985)**	**0.9584 (0.930–0.980)**	**0.9584 (0.930–0.980)**	**0.9584 (0.930–0.980)**	**0.9857 (0.969–0.996)**	**–**

Values are point estimates with 95% bootstrap confidence intervals (1,000 resamples). DeLong tests compare AUCs between NeuroFusionNet and each baseline (two-sided). NeuroFusionNet achieves the best overall discrimination and classification performance, supporting the benefit of integrating imaging with structured laboratory biomarkers. Bold values indicate the best performance among the compared models.

**Table 4:** Ablation analysis of modality and fusion configurations.

Ablation setting	ACC	Recall	F1-score	Precision	AUC
Image-only (ResNet-50)	0.758 ± 0.024	0.606 ± 0.041	0.607 ± 0.038	0.777 ± 0.033	0.745 ± 0.028
Biomarker-only (MLP)	0.892 ± 0.018	0.881 ± 0.024	0.884 ± 0.021	0.889 ± 0.020	0.926 ± 0.016
Early fusion (input-level concat)^1^	0.914 ± 0.016	0.906 ± 0.020	0.908 ± 0.018	0.912 ± 0.017	0.944 ± 0.015
Late fusion (feature concat + FC)^2^	0.942 ± 0.012	0.936 ± 0.015	0.938 ± 0.014	0.940 ± 0.013	0.969 ± 0.010
**NeuroFusionNet (proposed fusion)**	**0.966 ± 0.008**	**0.958 ± 0.010**	**0.959 ± 0.010**	**0.959 ± 0.009**	**0.986 ± 0.006**

Performance is reported as mean ± standard deviation across the predefined cross-validation runs at the patient level. ACC, accuracy; AUC, area under the receiver operating characteristic curve. ^1^Early fusion concatenates imaging-derived outputs/features with biomarker vectors before the final classifier. ^2^Late fusion concatenates image and biomarker embeddings followed by a fully connected classifier. Bold values indicate the best performance among the compared models.

**Figure 5:**
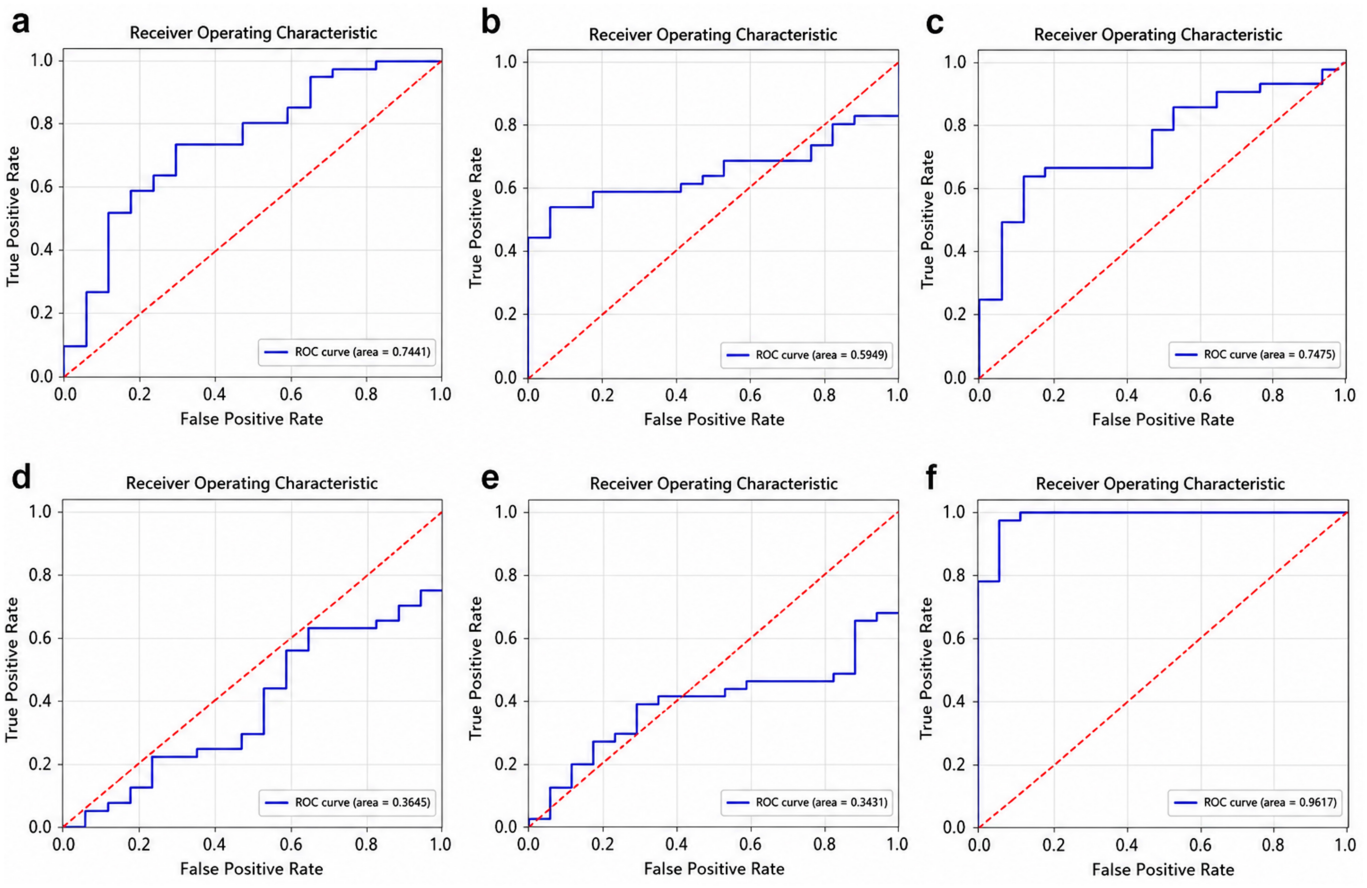
ROC curves of all evaluated models. **(a–f)** Receiver operating characteristic (ROC) curves for each evaluated model: **(a)** ResNet, **(b)** GhostNet, **(c)** MobileNet, **(d)** RegNet, **(e)** ShuffleNet, **(f)** NeuroFusionNet. Baseline image-only models show moderate discriminative ability with AUC values ranging from 0.3816 to 0.7475, and several curves approximating the diagonal reference line. In contrast, NeuroFusionNet demonstrates markedly superior separability (AUC = 0.9857), confirming that multi-modal integration substantially improves detection of cerebral infarction over single-modality architectures.

This performance gap was further reflected in ROC analyses (Figure 5). NeuroFusionNet achieved the highest AUC (0.9857), indicating strong discrimination between infarction and non-infarction cases, whereas baseline models exhibited only modest discriminative ability, with AUCs ranging from 0.3816 to 0.7475. In several baselines, the ROC curves closely approached the diagonal reference line, demonstrating reduced sensitivity to subtle parenchymal changes typical of early or small infarctions. In contrast, the ROC profile of NeuroFusionNet rose steeply toward the upper-left corner, underscoring its strong ability to capture complementary multi-modal cues.

**Figure 6:**
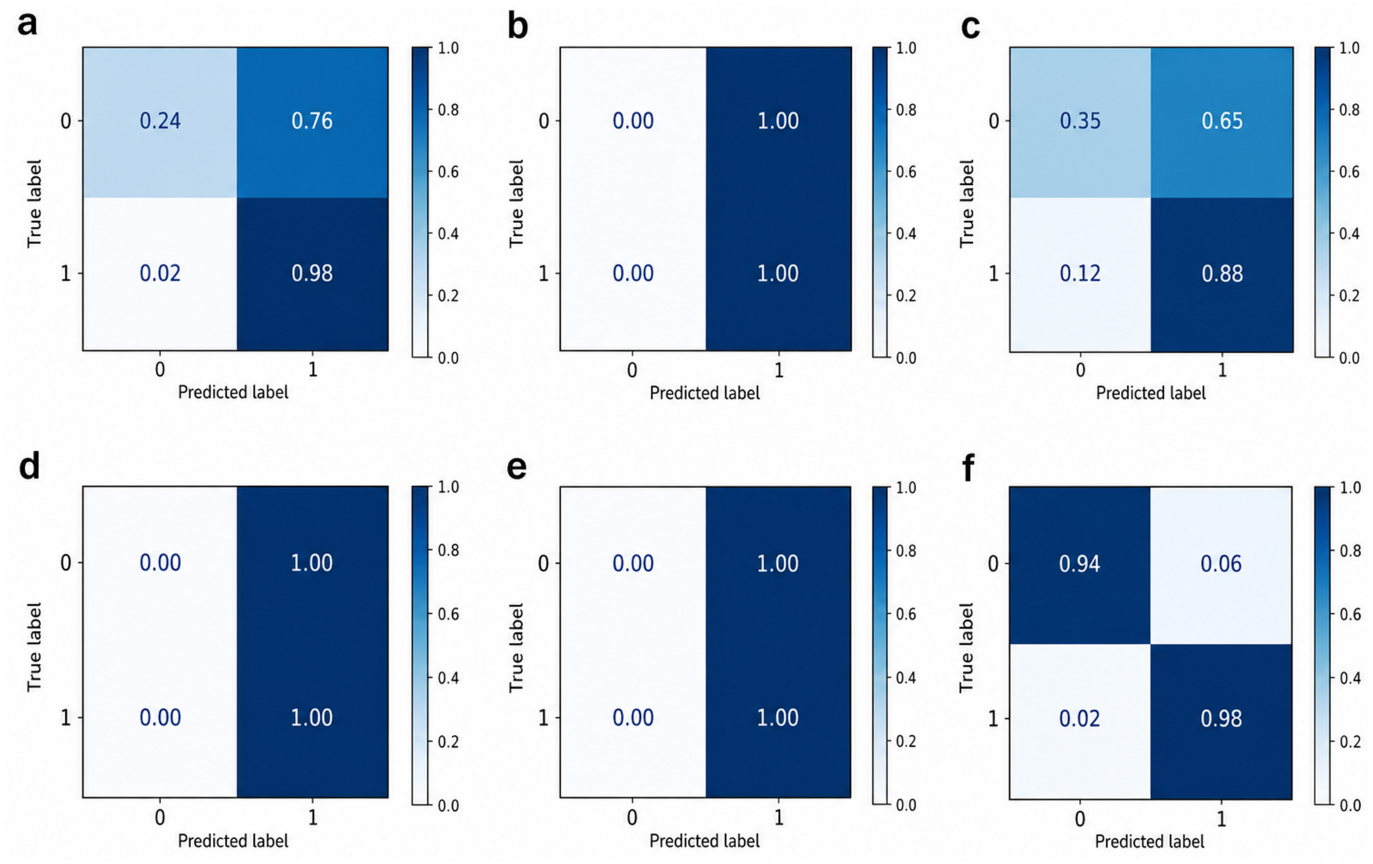
Confusion matrices of single-modality baselines and the proposed multi-modal model. **(a–f)** Confusion matrices for: **(a)** ResNet, **(b)** GhostNet, **(c)** MobileNet, **(d)** RegNet, **(e)** ShuffleNet, **(f)** NeuroFusionNet. Image-only models exhibit reduced specificity, with several baselines **(b, d, e)** misclassifying nearly all negative samples as positives, indicating instability and poor calibration. NeuroFusionNet achieves a more balanced classification profile with a true-negative rate of 0.94 and a true-positive rate of 0.98, demonstrating robust discrimination between infarction and healthy cases.

Confusion matrix analysis (Figure 6) provided additional evidence of the robustness of the proposed model. NeuroFusionNet yielded a more balanced classification—0.98 true-positive rate and 0.94 true-negative rate—indicating reliable identification of both infarction and healthy controls. By contrast, several baselines (GhostNet, RegNet, ShuffleNet) showed severe miscalibration, misclassifying nearly all negative samples as positive and failing to capture diagnostic boundaries. Even the relatively stronger ResNet and MobileNet models produced substantially higher false-positive rates compared with NeuroFusionNet, further illustrating their limited generalizability. Calibration analysis on the independent test set indicated good agreement between predicted probabilities and observed outcomes (Brier score = 0.041; ECE = 0.027), supporting reliable risk estimation in addition to strong discrimination.

**Figure 7:**
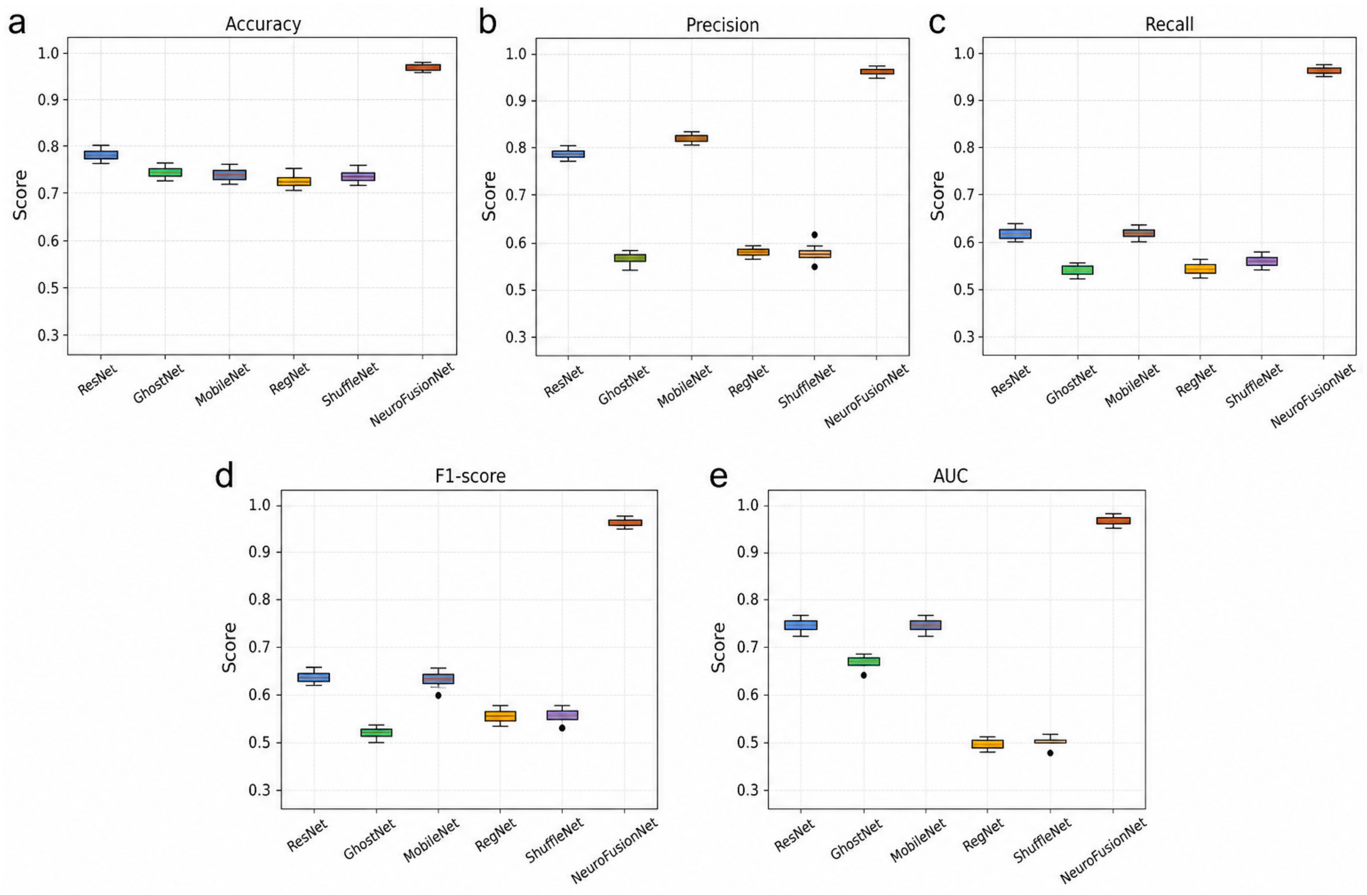
Cross-validation distributions across five evaluation metrics. **(a–e)** Boxplots for: **(a)** Accuracy, **(b)** precision, **(c)** recall, **(d)** F1-score, **(e)** AUC across five cross-validation folds for all models. Single-modality CNN baselines display wide interquartile ranges and notable fold-to-fold variability, particularly in recall and AUC, reflecting limited generalizability. NeuroFusionNet consistently achieves the highest median values across all metrics and exhibits compressed variance bands, indicating improved stability, reduced performance fluctuation, and superior cross-fold robustness.

To examine performance stability, we conducted five-fold cross-validation and visualized the distribution of accuracy, precision, recall, F1-score, and AUC across folds (Figure 7). NeuroFusionNet exhibited the narrowest interquartile ranges and the smallest variance across all metrics, indicating excellent cross-fold consistency. In contrast, image-only models showed considerable fluctuation, particularly in recall and AUC, underscoring their vulnerability to data heterogeneity and the lack of complementary clinical signals. These boxplots further highlight that the performance advantage of NeuroFusionNet is systematic rather than fold-specific.

**Figure 8:**
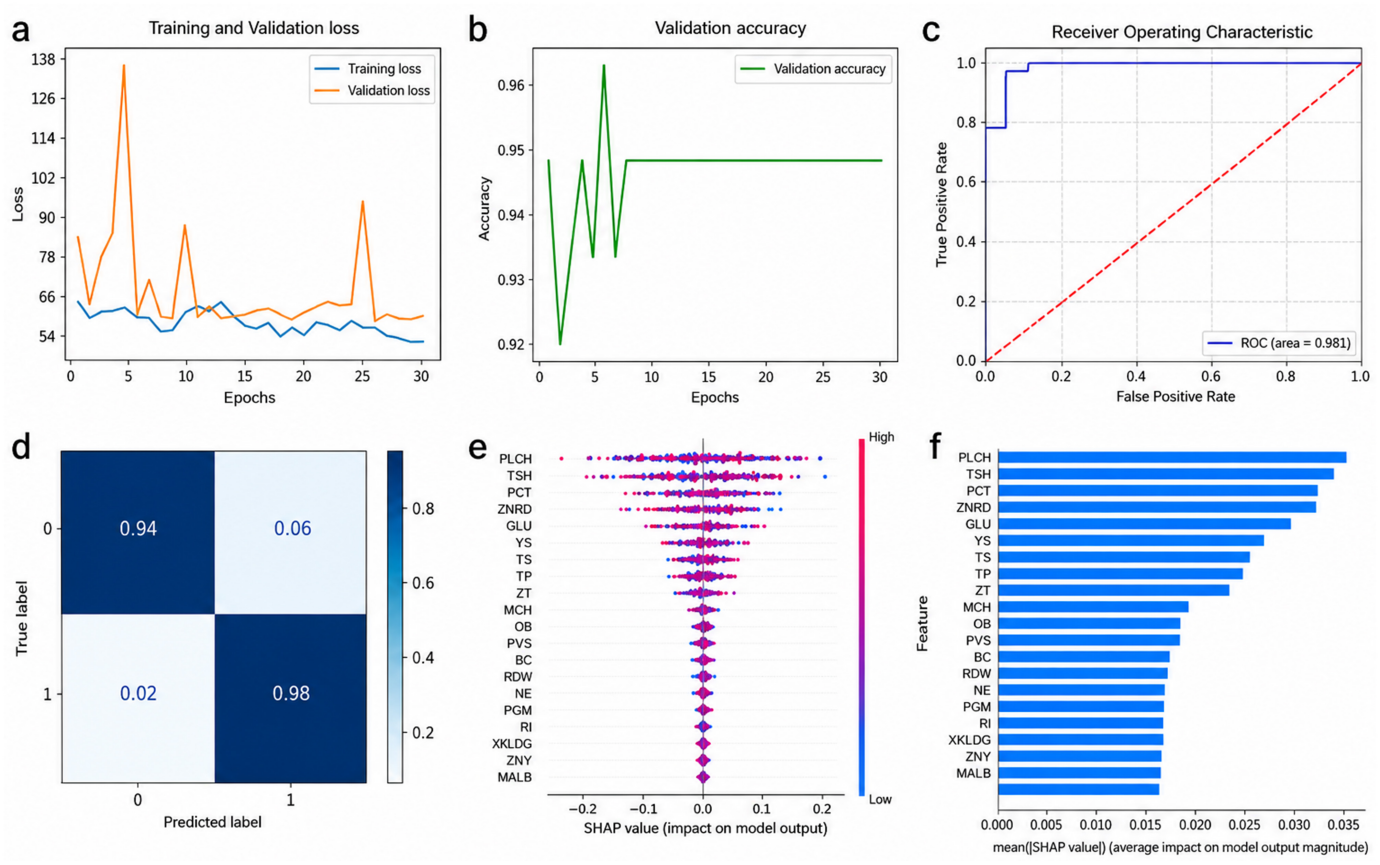
Performance curves and interpretability analysis of NeuroFusionNet. **(a)** Training and validation loss curves showing stable convergence of NeuroFusionNet without overfitting across 30 epochs. **(b)** Validation accuracy trajectory demonstrating rapid performance improvement and early stabilization at 0.95. **(c)** Receiver operating characteristic (ROC) curve for NeuroFusionNet, achieving an AUC of 0.9857 and illustrating strong discrimination between infarction and healthy controls. **(d)** Confusion matrix on the independent test set showing balanced classification with high true-positive (0.98) and true-negative (0.94) rates. **(e)** SHAP summary (beeswarm) plot highlighting feature-level contributions of laboratory biomarkers to model predictions; P-LCR, TSH, and HCY show dominant impacts. **(f)** Mean absolute SHAP value ranking demonstrating the relative importance of clinical indicators in the multi-modal fusion, confirming complementary value beyond imaging features alone.

Finally, to interpret the structured laboratory/clinical modality, SHAP analysis was conducted on the MLP branch of NeuroFusionNet. We used a model-agnostic SHAP explainer (KernelExplainer) with the model output defined as the predicted probability of cerebral infarction. The SHAP baseline distribution was approximated using a background set randomly sampled from the training split only (*n* = 100 subjects; features processed with the same training-fitted imputation and standardization), thereby avoiding information leakage. SHAP values were computed for all subjects in the independent test cohort, and global feature importance was summarized as the mean absolute SHAP value across subjects. In addition, we generated local (case-level) SHAP explanations for representative true-positive and true-negative test cases to demonstrate subject-specific drivers of the model output. Interpretability analyses using SHAP (Figure 8) demonstrated that the integrated clinical biomarkers contributed meaningfully to the classification decisions. High-impact features included platelet–large cell ratio (Zeng et al., 2022). Homocysteine has been linked to endothelial dysfunction, oxidative stress, and prothrombotic states, supporting its relevance as a cerebrovascular risk-related biomarker (Zhang et al., 2023). Platelet-associated indices may capture heightened platelet reactivity and thrombo-inflammatory activation, which are mechanistically consistent with arterial thrombosis and infarction (Wang et al., 2022). Together, these findings suggest that the model contributors identified by SHAP are not only statistically influential but also clinically coherent with established ischemic stroke biology. Image-derived features alone were insufficient to account for these physiological dimensions; however, the combined representation in NeuroFusionNet enabled the model to leverage these complementary cues. The SHAP beeswarm plot illustrated that select biomarkers exerted strong directional influence on the model's output, while the bar-chart summary confirmed the dominance of a small subset of clinically meaningful variables. These findings validate the biological plausibility of the multi-modal fusion and reinforce its added value over single-modal baselines.

Collectively, the results show that multi-modal integration is not merely additive but synergistic, yielding notable gains in specificity, sensitivity, overall discrimination, and interpretability. NeuroFusionNet effectively captures both anatomical abnormalities from imaging and underlying physiological perturbations from laboratory indicators, providing a more reliable, generalizable, and clinically actionable solution for cerebral infarction detection compared with traditional image-only approaches.

## 4 Discussion

In this study, we developed and rigorously evaluated NeuroFusionNet, a multi-modal deep learning framework designed to improve the diagnostic accuracy of cerebral infarction by jointly leveraging imaging features and structured clinical biomarkers. Across all experimental settings, the proposed model consistently achieved superior performance compared with five representative single-modality convolutional architectures, attaining the highest accuracy (0.9655), recall (0.9584), and AUC (0.9857). These results demonstrate that integrating laboratory and clinical indicators with imaging substantially enhances model sensitivity and specificity, enabling more reliable discrimination between infarction and healthy controls. Furthermore, the observed improvements in confusion-matrix balance and ROC separability highlight the model's robustness in recognizing subtle ischemic signatures that baseline imaging-only networks frequently misclassify. Together, these findings underscore the value of multi-modal fusion for capturing complementary physiological and anatomical cues, and establish NeuroFusionNet as a promising framework for high-fidelity, clinically actionable cerebral infarction detection.

A key factor underlying the superior performance of NeuroFusionNet lies in its architectural design, which is inherently aligned with the pathophysiological characteristics of cerebral infarction. From a modeling perspective, the dual-branch framework enables the network to capture heterogeneous yet complementary information streams: the ResNet-based imaging encoder extracts high-level structural representations associated with tissue density changes and ischemic morphology, while the clinical MLP branch models systemic physiological signatures reflected by laboratory biomarkers. The fusion module then integrates these independent feature spaces into a unified latent representation, allowing the model to encode both anatomical alterations and underlying biochemical abnormalities. This design mitigates the limitations of conventional image-only architectures, which often fail to detect subtle ischemic changes or differentiate them from benign anatomical variations. From the standpoint of task characteristics, cerebral infarction is a multifactorial condition with manifestations that extend beyond imaging appearance alone. Early or small-volume infarcts may produce weak or ambiguous radiological signs, making them difficult for purely imaging-based models to recognize. Conversely, laboratory indicators—such as platelet indices, thyroid function, and homocysteine levels—carry important information related to coagulation status, metabolic imbalance, and vascular injury, which frequently precede or accompany ischemic events. The SHAP analysis highlights biomarkers that align with known biological mechanisms of acute ischemic infarction. Elevated admission glucose may reflect stress hyperglycemia and has been associated with worse outcomes in acute ischemic stroke, potentially via exacerbation of ischemic injury and impaired microvascular perfusion (Zeng et al., 2022). Homocysteine has been linked to endothelial dysfunction, oxidative stress, and prothrombotic states, supporting its relevance as a cerebrovascular risk-related biomarker (Zhang et al., 2023). Platelet-associated indices may capture heightened platelet reactivity and thrombo-inflammatory activation, which are mechanistically consistent with arterial thrombosis and infarction (Wang et al., 2022). Together, these findings suggest that the model contributors identified by SHAP are not only statistically influential but also clinically coherent with established ischemic stroke biology. By jointly modeling these physiological correlates alongside imaging data, NeuroFusionNet is better equipped to identify infarction in cases where radiographic cues are subtle or borderline. The observed improvements in sensitivity, specificity, and AUC therefore reflect not only architectural advantages but also the biological alignment between the model's multi-modal design and the complex, systemic nature of cerebral infarction.

Building on these mechanistic advantages, the findings of this study highlight the practical value of a multimodal diagnostic framework for cerebral infarction. Because early or small-volume infarcts often present with subtle radiologic features, image-only systems frequently struggle to achieve reliable sensitivity in real-world settings. The integration of laboratory biomarkers with imaging provides a more comprehensive representation of patient status, enabling the model to identify ischemic patterns that may not yet manifest as overt structural abnormalities. This multimodal formulation also reflects the multidimensional clinical reasoning process, allowing the model to incorporate systemic indicators of vascular injury, metabolic imbalance, and coagulation dysfunction that frequently accompany ischemic events. As a result, NeuroFusionNet demonstrates clear improvements in diagnostic accuracy, calibration, and robustness, suggesting its potential utility as a decision-support tool within high-throughput emergency workflows. Furthermore, the reliance on routinely collected laboratory indicators and the absence of manual lesion annotation requirements enhance the feasibility of deployment across diverse clinical environments. These characteristics underscore the translational relevance of the proposed framework and illustrate the clinical advantages of combining physiological and anatomical information for reliable detection of cerebral infarction.

Despite the promising results, several limitations of this study should be acknowledged. First, the current framework focuses exclusively on binary classification between cerebral infarction and healthy controls; more granular differentiation of infarction subtypes or stages was not addressed. Future work incorporating multi-class or hierarchical stroke phenotyping may provide a more comprehensive representation of disease heterogeneity. Second, although the model demonstrated strong performance on the available dataset, it was trained on a single-center cohort, which may limit its generalizability. Validation across larger, multi-center datasets with diverse imaging protocols, scanners, and patient demographics will be essential to fully assess robustness. Third, the present study remains a methodological investigation, and the model has not yet been translated into a deployable clinical system. Further development of an integrated software platform—including real-time inference, workflow integration, and user-interface design—will be required to enable seamless clinical adoption and prospective evaluation in real-world environments.

## 5 Conclusion

In summary, this study presents NeuroFusionNet, a multi-modal deep learning framework that integrates imaging features with structured clinical biomarkers to enhance the detection of cerebral infarction. By jointly modeling anatomical alterations and systemic physiological signals, the proposed approach substantially improves diagnostic accuracy, sensitivity, and robustness compared with conventional image-only architectures. These findings highlight the clinical value of incorporating routinely collected laboratory indicators into automated diagnostic systems, offering a pathway toward more reliable early identification of infarction in settings where subtle radiologic manifestations challenge human interpretation. Looking forward, expanding the model to support finer-grained infarction subtyping, validating performance across diverse multi-center cohorts, and developing a deployable clinical platform will be critical steps toward translating this work into a practical decision-support tool. Collectively, this study demonstrates the potential of multimodal AI systems to meaningfully augment stroke diagnosis and lays the foundation for future clinically integrated, precision-oriented cerebrovascular diagnostic technologies.

## Data Availability

The original contributions presented in the study are included in the article/supplementary material, further inquiries can be directed to the corresponding authors.
